# Algicidal bacteria trigger contrasting responses in model diatom communities of different composition

**DOI:** 10.1002/mbo3.818

**Published:** 2019-02-27

**Authors:** Arite Bigalke, Georg Pohnert

**Affiliations:** ^1^ Institute for Inorganic and Analytical Chemistry, Bioorganic Analytics Friedrich Schiller University Jena Jena Germany; ^2^ Max Planck Institute for Chemical Ecology Jena Germany

**Keywords:** algicidal bacteria, co‐cultures, community ecology, diatoms, phytoplankton, Plankton interactions

## Abstract

Algicidal bacteria are important players regulating the dynamic changes of plankton assemblages. Most studies on these bacteria have focused on the effect on single algal species in simple incubation experiments. Considering the complexity of species assemblages in the natural plankton, such incubations represent an oversimplification and do not allow making further reaching conclusions on ecological interactions. Here, we describe a series of co‐incubation experiments with different level of complexity to elucidate the effect of the algicidal bacterium *Kordia algicida* on mixed cultures of a resistant and a susceptible diatom. The growth of the resistant diatom *Chaetoceros didymus* is nearly unaffected by *K. algicida* in monoculture, while cells of the susceptible diatom *Skeletonema costatum* are lysed within few hours. Growth of *C. didymus* is inhibited if mixed cultures of the two diatoms are infected with the bacterium. Incubations with filtrates of the infected cultures show that the effects are chemically mediated. In non‐contact co‐culturing we show that low concentrations of the lysed algae support the growth of *C. didymus*, while higher concentrations trigger population decline. Complex cascading effects of algicidal bacteria have thus to be taken into account if their ecological role is concerned.

## 1 INTRODUCTION

Microalgae of the phytoplankton can form spatially and temporally limited blooms within the pelagic environment. Such blooms can be limited by abiotic factors, such as light or nutrient availability. However, a recent survey reflects that environmental factors are insufficient predictors of community structures (Lima‐Mendez et al., 2015). Additional biotic interactions can lead to species dominance and bloom decline. Mechanisms mediating decline of algal populations include grazing by herbivores and viral infection as well as allelopathic interactions in which growth of microalgae is chemically suppressed by competitors (Bratbak, Egge, & Heldal, 1993; Brussaard et al., 1995; Pohnert, 2010). Cell‐lysis caused by algicidal bacteria is another factor that can substantially influence the plankton community (Bidle, 2015; Meyer, Bigalke, Kaulfuss, & Pohnert, 2017). The heterotrophic bacteria can utilize resources excreted by phytoplankton cells or resources released after algal cell death and lysis (Bidle & Falkowski, 2004; Meyer et al., 2017). Due to their universal distribution, algicidal bacteria are of major interest for the understanding of dynamic species successions in the ocean (Teeling et al., 2016; van Tol, Amin, & Armbrust, 2017). However, research on this class of organisms is mainly limited to investigations of bilateral interactions of one bacterial isolate with a specific phytoplankton species (Meyer et al., 2017). In nature, the situation is clearly more complex since multiple players form complex interaction networks that can be disturbed by lytic bacteria. Bacteria might, for example, target one single species within a community by specific lysis or eliminate multiple members of the phytoplankton thereby making room for successive blooms (Meyer et al., 2017). Algae that show resistance against such generalist algicidal bacteria might, therefore, have a substantial competitive advantage that could boost their performance in succession of a lytic event.

To study such potential cascading effects, we set up a tripartite interaction network including a resistant and a susceptible diatom species that were exposed to the algicidal bacterium *Kordia algicida*. We focused on the two widely distributed diatoms *Skeletonema costatum*, which is susceptible to bacterial lysis and the resistant *Chaetocerous didymus *(Paul & Pohnert, 2011). Both diatoms are globally distributed and co‐occur as succeeding bloom forming algae in the environment (Kooistra et al., 2008; Li et al., 2017). It has been recently established in our lab that the algicidal activity of *K. algicida *is based on the release of proteases that are under the control of quorum sensing mediators (Paul & Pohnert, 2011). The resistance of *C. didymus *involves the induced release of a presumably counteracting protease (Paul & Pohnert, 2013) confirming that chemical cues are the primary language used by marine organisms (Hay, 2009). To investigate how chemically mediated interactions might be involved in further cascading effects following algal lysis we address here mixed co‐cultures, as well as the activity of filtrates of infected and non‐infected cultures on the respective interaction partners. Additionally, induction of responses that are triggered by diffusible chemical mediators was investigated in non‐contact co‐culturing experiments that allow the free diffusion of chemical signals in between culture compartments containing the respective partners (Paul, Mausz, & Pohnert, 2012). We clearly establish that combinations of only two species in simple laboratory setups do not allow to predict algal performance upon infection in more complex settings. Despite the intrinsic problematic transfer from laboratory to field scenarios our results suggest that in nature lytic bacteria might cause unexpected cascading effects. We also suggest that care has to be taken when planning to use such bacteria in the control of harmful algal blooms since these unpredictable events might lead to even more harmful scenarios.

## 2 MATERIALS AND METHODS

### 2.1 Diatom and bacteria culturing

*S. costatum* (RCC75) was obtained from the Roscoff Culture Collection France and *C. didymus* (Na20B4) was obtained from Wiebe Kooistra who isolated it in the Gulf of Naples, Italy (LTER sample station Marechiara). Both non‐axenic diatoms were grown in artificial sea water (SW) prepared according to Maier and Calenberg at a pH of 7.8 (Maier & Calenberg, 1994) under a 14/10 hr light/dark cycle with 50–60 µmol m^−2^ s^−1^ at 13°C. The initial nutrient concentrations were 620 mM nitrate, 14.5 mM phosphate, and 320 mM silicate. Algal growth was determined either by measuring the relative *in vivo* chlorophyll *a* fluorescence on a Mithras LB 940 plate reader (Berthold Technologies, Bad Wildbad, Germany) using 200 µl of each culture in dark 96‐well plates or by cell counting after fixation with Lugol´s iodine solution using Fuchs‐Rosenthal counting chambers under an upright microscope (Leica DM 200, Wetzlar, Germany).

The Gram‐negative marine bacterium *K. algicida* strain OT‐1 was obtained from the NITE Biological Resource Center (NBRC 100336) and stored at −80°C in 20 vol% glycerol. Growth of *K. algicida* was maintained at 23°C and controlled by measuring the optical density (OD) at a wavelength of 550 nm (Specord M42 UV‐vis spectrophotometer by Carl Zeiss, Jena Germany). Bacterial inoculation densities were set to a final OD_550_ of 0.02 for all the experiments. For experiments 1–3, *K. algicida* was grown on solid full medium plates (3.74% w/v marine broth and 1.5% w/v agar) for three days before harvesting. The inoculation solution for treatments in the experiments was obtained by washing off the bacterial cells from the plates with SW. Inoculation procedure for experimental controls was identical but plates without bacterial cells were used instead.

### 2.2 Experiment 1

We initially tested how the bacterial lysed *S. costatum* affects the success of the resistant diatom *C. didymus* in mixed cultures. Two independent fully replicated sets of experiments were carried out with *C. didymus* and *S. costatum* bialgal cultures at an initial cell density of 1×10^3^ and 1×10^5^ cells/ml, respectively, in presence or absence of the algicidal bacterium. Eighty milliliters of algal dilutions were cultivated as described above in cell culture flasks (T‐75, Sarstedt, Nümbrecht, Germany). Monoalgal controls were conducted in the second experiment. *K. algicida* was cultivated and inoculated as described above. Data points were obtained by cell counts resulting in total replicates of four to seven from both experiments. We used non‐parametric Mann–Whitney Rank Sum tests (U‐test) to obtain significant differences in the contact cultures (Table A1a).

### 2.3 Experiment 2

We assessed the effects of bacterial lysed *S. costatum* on the growth of *C. didymus* by exposing exponential precultured *C. didymus* to filtrates of *S. costatum* in declining phase and *S. costatum* lysed by *K. algicida* at three successive time points after bacterial inoculation. *S. costatum* monocultures (100 ml) were inoculated (*n* = 6) into cell culture flasks (T‐75, Sarstedt). *S. costatum* growth was assessed as *in vivo* chlorophyll *a* fluorescence (RFU) and *K. algicida* was introduced at the beginning of declining phase of *S. costatum* as described in “diatoms and bacteria culturing”. Filtrates were obtained directly after *K. algicida* inoculation (day 0) and after five and 10 days, respectively, by processing the cultures in the following order: gentle centrifugation (3 min.; 570*g*; Hermle Z400, Wehingen, Germany), filtration through membranes with a pore size of 5 µm (13 mm, Nucleopore Track‐Etch membrane, Whatman, Kent, UK) placed in a syringe filter holder (13 mm, Swinnex®, Merck, Darmstadt, Germany). Subsamples of 5 µm filtrates were further sterile filtered (syringe filter unit Filtropur S 0.2 µm with a polyethersulfone (PES) membrane, Sarstedt).

Two hundred and fifty microliters of either 5 µm filtrates, 0.2 µm filtrates or sea water (control) were immediately added to 250 µl *C. didymus* cultures (each in independent triplicates) and incubated in 24‐well plates. *C. didymus* growth was followed by measuring *in vivo* chlorophyll *a* fluorescence (RFU). These triplicates were averaged before data processing. The average of sea water control was used as reference value (see below). *C. didymus* growth rate was calculated from day 0 to day 3: µ = ((ln (n_3_/n_0_))/t), where n_3_ and n_0_ refers to chlorophyll *a* fluorescence at day 3 and day 0, respectively. The effects of filtrates obtained from *S. costatum* cultures on *C. didymus* were normalized as per cent growth relative to control: (µ[_trt_]/µ[_ctrl_]) * 100 where µ[_trt_] is the growth rate of *C. didymus* exposed to the 5 µm or 0.2 µm‐filtrates of the competing diatom and µ[_ctrl_] is the growth rate of *C. didymus* exposed to the respective sea water control.

Bacterial algicidal activity (Paul & Pohnert, 2011) was indirectly assessed by protease activity of the sterile filtrates measured immediately at each medium sampling time point using a commercial Protease Assay Kit (EnzChek™, Invitrogen, Carlsbad CA, USA) which is based on the conversion of a casein dye to a fluorescent product (Jones et al., 1997). The assay was performed according to manufactures instructions and as described previously (Paul & Pohnert, 2011). Briefly, 10 µl of cell‐free filtrates were diluted in 100 µl digestion buffer and 100 µl of the dye at a concentration of 10 µg per ml were added. Samples were incubated in the dark at room temperature for one hour prior to fluorescence read out on a Mithras LB plate reader with an excitation wavelength of 470 ± 5 nm and an emission wavelength of 510 ± 20 nm.

We used unpaired two‐sided *t* tests to obtain significant differences in chlorophyll *a* units (RFU) for *S. costatum* growth comparison and relative growth rate comparisons (%) for *C. didymus *(Table A2aa,c). Protease activities (RFU) were compared using One way Analysis Of Variance (ANOVA) followed by Holm–Sidak *post hoc *test when significant differences occurred (Table A2ab).

### 2.4 Experiment 3

Experiment 3 was conducted with co‐cultivation chambers in a non‐contact situation to further determine whether the ability to alter *C. didymus* success is chemically mediated or relies on the contact of *C. didymus* with *S. costatum* and its associated bacteria. The co‐culture setup consists of two glass vessels separated by a 0.22 µm hydrophilic polyvinylidene fluoride (PVDF) membrane (Durapore, Millipore, Billerica, MA, USA) which allows exchange of small molecules but blocks the passage for all cells (Paul et al., 2012). Modifying the procedure from (Paul et al., 2012) we used a modified co‐cultivation setup with size‐reduced chambers leading to ten times less inoculation volume per vessel. Each vessel can hold 50 ml, has a 43 mm flat edge opening and a 15 mm opening for filling and sampling. Both vessels are fitted together by four screws fixing two fastening rings one at each chamber. Each co‐culture contains both, 50 ml of late exponential or early stationary precultured *S. costatum* (Sc) at either initially 7 × 10^4^ cells/ml (low‐Sc) or 2 × 10^5^ cells/ml (high‐Sc) concentration in one compartment and 50 ml of exponential precultured *C. didymus* (Cd) at initial cell densities of 1 × 10^4^ cell/ml in the other. *K. algicida* (Ka) was introduced as described under “diatoms and bacteria culturing” into each of the diatom containing vessels. All setups were cultivated under conditions described in “diatoms and bacteria culturing”, however with constant slow shaking. Growth was measured as *in vivo* chlorophyll *a* fluorescence (RFU) in five (low‐Sc/Cd, high‐Sc/Cd, low‐Sc+Ka/Cd+Ka, high‐Sc+Ka/Cd+Ka) replicates, respectively. We used an unpaired two‐sided *t* test to test for significant differences in algal growth (RFU) (Table A3).

### 2.5 Experiment 4

Directly following the last measurement of the co‐culture experiment (day 11), the cultures were tested for growth of *K. algicida*, the strain used for inoculation at the beginning of the co‐cultivation. Total remaining volumes of each co‐culture (both compartment) were gently vacuum filtered through a 1.2 µm GF/C, 47‐mm filter (Whatman) before sterile filtration (syringe filter unit Filtropur S 0.2, Sarstedt). Two filtrates of “high‐Sc/Cd” replicates were accidentially discarded during processing and not used for *K. algicida* inoculations. Three (high‐Sc/Cd) and five (low‐Sc+Ka/Cd+Ka, high‐Sc+Ka/Cd+Ka, and low‐Sc/Cd) replicates corresponding to the replicates during co‐cultivation were used for bacterial inoculations. Twelve milliliters of the obtained sterile solutions were transferred to culture flasks and inoculated with a *K. algicida* culture (final OD_550_ of 0.02) previously starved for three days in SW after stationary growth in liquid minimal medium, containing 10 amino acids (aspartic acid, alanine, glutamic acid, glycine, isoleucine, leucine, methionine, phenylalanine, proline, and valine in a final concentration of each 0.08% w/v) to reduce carry over from the full medium. Control media for the experiment used were SW and a minimal medium (each *n* = 1) containing six amino acids (aspartic acid, glutamic acid, glycine, leucine, methionine, and valine in a final concentration of each 0.08% w/v). Growth of *K. algicida* was maintained at 23°C during constant shaking and was followed over eight days by optical density (550 nm) measurements. We used non‐parametric Mann–Whitney Rank Sum tests (U‐test) to obtain significant differences in treatments from the co‐culture system (Table A4).

### 2.6 Data analysis

Statistical analysis was conducted using SigmaPlot 13.0 software (Systat Software Inc., London, UK). Levels of significance are given as *p < 0.05, **p < 0.01, and ***p < 0.001. *p* > 0.05 is considered as not significant.

## 3 RESULTS

### 3.1 Effect of *K. algicida*‐induced lysis of *S. costatum *on the growth of *C. didymus* in mixed cultivation (Experiment 1)

**Figure 1:**
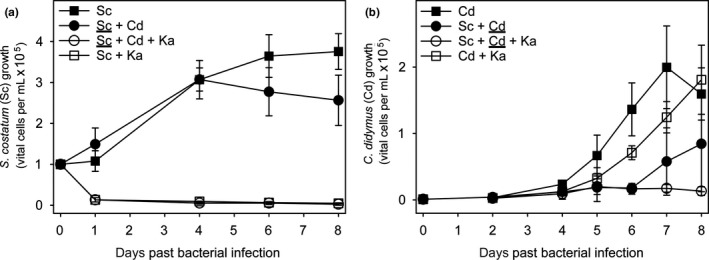
Diatom growth in tripartite mixed cultivation. Cell numbers of (a) *S. costatum* (Sc) and (b) *C. didymus* (Cd) with or without additions of *K. algicida* (Ka) in mono‐ or bialgal contact co‐cultivation. Results are expressed as the mean of four to seven replicates ± *SD*. Non‐parametric statistical tests were used for comparisons (Data about the statistical evaluation are given in Table A1aa,b). The underlined species in mixed cultivation is the one for which the cell numbers are given

**Figure A1:**
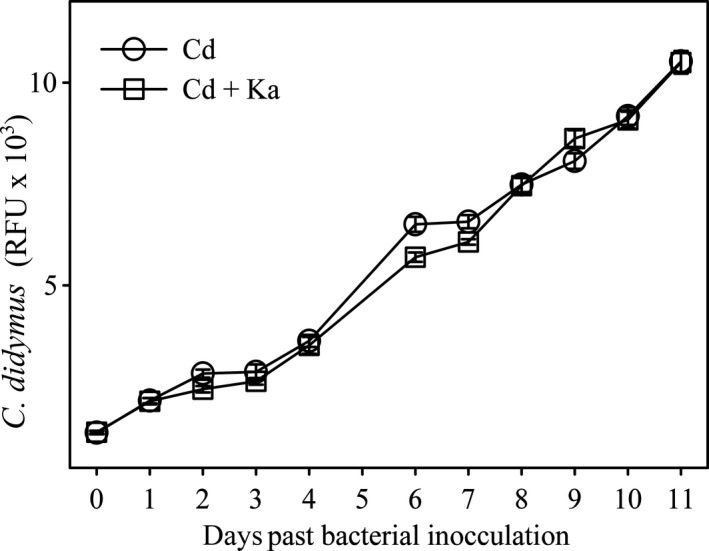
Growth of *C. didymus* (Cd) with and without added *K. algicida* (Ka). Results are expressed as the mean of five replicates ± *SD*. Conditions were otherwise as in Figure 1 of the main manuscript

Before inoculation with bacteria, the initial cell abundance ratio of *S. costatum* to that of *C. didymus* was 100:1 to compensate the higher growth rate and larger cell volumes of *C. didymus*. At these cell densities, *S. costatum* growth is lower in co‐cultures with *C. didymus* compared to the control (Figure 1a). This effect was significant on day 8 (*p* = 0.038). In the presence of *K. algicida*, *S. costatum* is quantitatively lysed on day 1 and the (co‐)cultures do not recover. Both *K. algicida* treatments have significant negative effects on *S. costatum* compared to the respective controls from day 1 onwards (Table A1aa). The presence of *C. didymus* did not affect the overall lysis, indicating that no protective effect in co‐cultures can be observed (*p* > 0.1 at all days after inoculation except day 4 Table A1aa). Growth responses of *C. didymus* in mixed cultivation with *S. costatum *and/or *K. algicida *are shown in Figure 1b. *S. costatum* in the co‐culture delayed the growth of *C. didymus* from the fifth day onwards (*p* ≤ 0.038, Table A1ab); however, this effect was not significant on the last day of the experiment (*p* = 0.257). *C. didymus* is delayed only slightly (significant only on day 4 and 6, Table A1ab) in the presence of *K. algicida *and reaches comparable cell counts at day 8, indicating resistance of the algae*. *This was confirmed in an independent experiment where no significant effect of *K. algicida *on *C. didymus *was observed (Figure A1). *C. diymus* growth is fully inhibited when the co‐cultured *S. costatum* was lysed by the algicidal bacterium (*p* = 0.001 at day 8 compared to cell abundances in bialgal cultivation; *p* ≤ 0.042 from day 5 onward compared to *C. didymus* +K*. algicida*). Taken together, both diatoms exhibit an inhibitory effect on each other in co‐culture. *K. algicida *quantitatively lyses *S. costatum* and the combination of lysed cells and *K. algicida* arrests growth of *C. didymus*.

### 3.2 Effects of filtrates of *S. costatum *cultures that were lysed with *K. algicida *on growth of *C. didymus* (Experiment 2)

**Figure 2:**
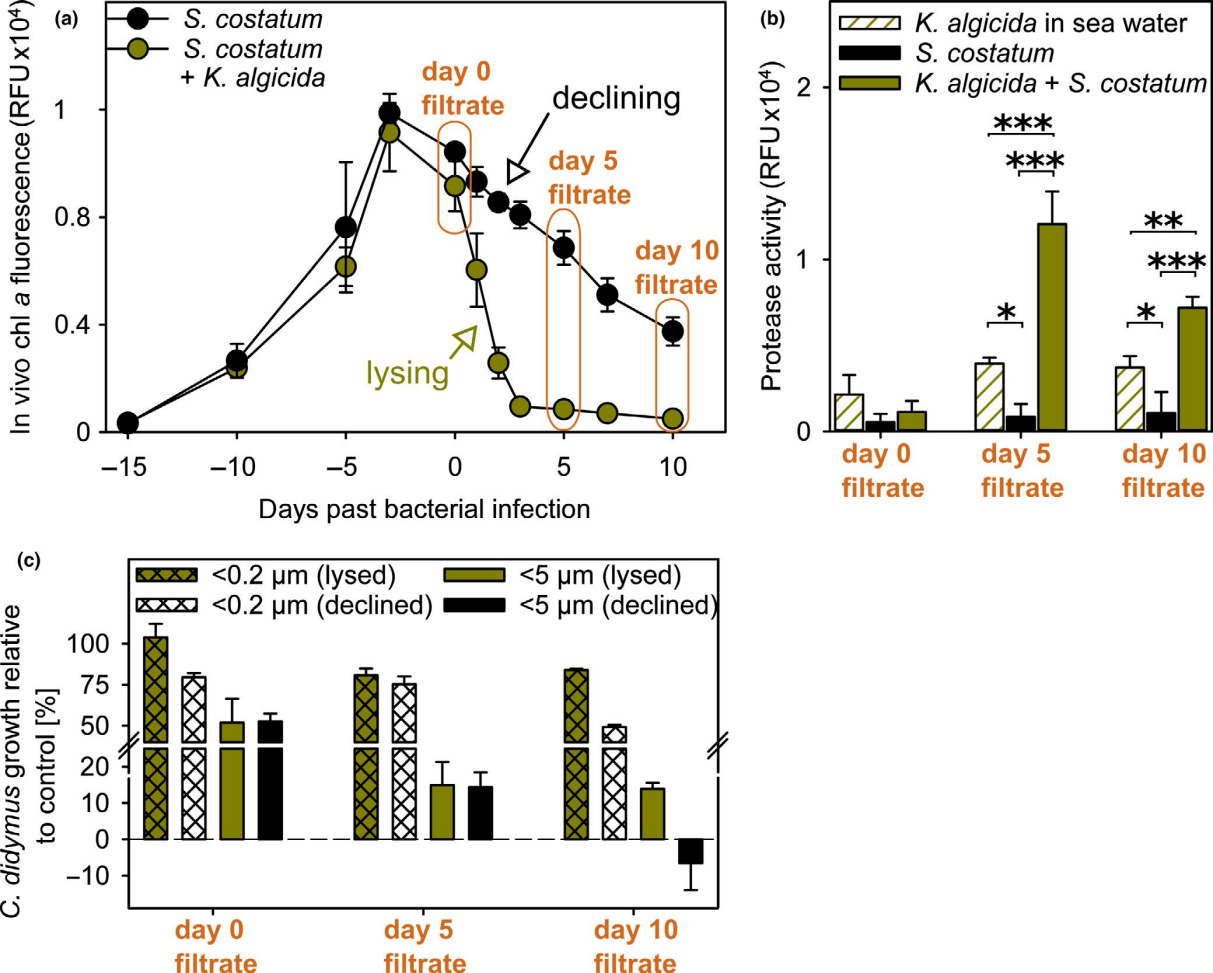
Impact of declining and bacterial‐lysed *S. costatum* culture filtrate on *C. didymus*. (a) Growth curve of *S. costatum* (± *K. algicida* infection at day 0) (both *n* = 3 ± *SD*). Orange sections indicate the time when filtrate was harvested and further processed by filtration (5 µm and 0.2 µm). Unpaired *t* tests were performed to obtain significant differences in growth (Table A2aa). (b) Protease activity of *K. algicida* infected *S. costatum* filtrates (*n* = 3) compared to filtrates of *K. algicida* in sea water (technical triplicates, one duplicate) and declining *S. costatum* cultures (*n* = 3). Background level of protease activity (sea water control) were substracted before plotting and statistical analysis (One way of variance analysis (ANOVA), Table A2ab). Asterisks show significant differences compared to the controls. Levels of significance are given as *p < 0.05, **p < 0.01, and ***p < 0.001. (c) Inhibitory activity of filtrates from *S. costatum* and infected *S. costatum *on *C. didymus* growth. Relative growth of *C. didymus* (% RFU of respective control) after three days of incubation (*n* = 3 ± *SD*) was tested for significant differences using unpaired *t* tests (Table A2ac)

*K. algicida* lyses stationary to declining *S. costatum* cultures within one day (*p* = 0.018) that then remain significantly suppressed until day 10 (*p* < 0.001, Table A2aa) (Figure 2a). Five and 10 days after *K. algicida*‐induced lysis of *S. costatum*, protease activity is significantly increased compared to the non‐infected but declining *S. costatum* cultures (both *p* < 0.001, Table A2ab) and also compared to *K. algicida* controls (both *p* ≤ 0.006) (Figure 2b).

Filtrates of *K. algicida*/*S. costatum* co‐cultures as well as of pure bacteria and diatom cultures were obtained on the day of inoculation (day 0 filtrate), as well as on day 5 (day 5 filtrate) and day 10 (day 10 filtrate) after inoculation. Two types of filtrates using different pore‐sized filters (5 µm and 0.2 µm, respectively) were generated and administered to *C. didymus* cultures. The growth of *C. didymus* was monitored after three days and is given as growth (%) relative to unialgal *C. didymus* controls in sea water (Figure 2c).

Filtrate (<5 µm) from declining *S. costatum* cultures inhibits *C. didymus* growth already at the start of the experiment (Figure 2c, gray). At later days of the experiment with progressing decline of the culture, this effect becomes more pronounced. Filtrate from day 10 of non‐infected *S. costatum* inhibits *C. didymus* growth fully. Throughout the experiment, the <5 µm filtrates were more active compared to the <0.2 µm filtrates (*p* ≤ 0.001 for all incubations, Table A2ac). Filtrates (<5 µm) from *S. costatum* cultures that were lysed by *K. algicida *caused also pronounced inhibition of *C. didymus *growth (Figure 2c, green). Again the <5 µm filtrate was more active compared to the <0.2 µm filtrates (*p* ≤ 0.006 for all incubations, Table A2ac). Comparison of the impact of *S. costatum* filtrates in declining phase to filtrates from bacterial‐ lysed cultures shows that the diatom was generally less affected when the bacterium was present, which was significant with both types of day 10 filtrates (significant for both incubations, Table A2ac) and with <0.2 µm day 0 filtrate (*p* = 0.008). The increased protease activity in *K. algicida* lysed cultures (Figure 2b) has thus no negative effect on *C. didymus*. In summary, filtrate from declining *S. costatum* cultures, whether bacterial‐lysed or not, exhibits a strong negative allelopathic effect on *C. didymus*. *K. algicida* alleviates this effect partially.

### 3.3 Effect of *K. algicida*‐induced lysis of *S. costatum *on the growth of *C. didymus* in non‐contact co‐cultivation (Experiment 3)

**Figure 3:**
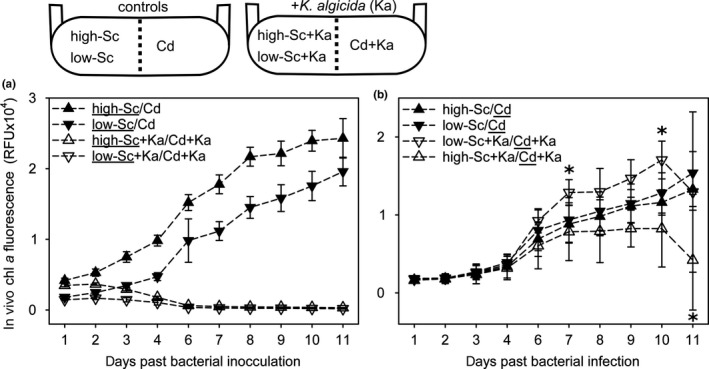
Diatom growth in tripartite non‐contact co‐cultivation. Growth development of (a) *S. costatum* (Sc) and (b) *C. didymus* (Cd) with or without *K. algicida* (Ka) infection in non‐contact co‐cultivations. The underlined species in mixed cultivation is the one for which the cell numbers are given. Results expressed as mean of five replicates ± *SD* with asterisk indicating significant differences (*p* < 0.05, unpaired *t* test, Table A3)

Experiment 2 demonstrated that the interaction of *C. didymus* with *S. costatum* is at least partly chemically mediated. Testing filtrates, as in Experiment 2 does not allow to conclude about additional dynamic mechanisms influencing the interaction. These could include the induction of chemical responses by signaling molecules or by modulated resource activity. To learn more about these aspects, we conducted co‐cultivation experiments where the diatoms are physically separated by a membrane that allows the diffusion of chemical signals (Figure 3). We included treatments with comparable *S. costatum* cell counts to those in Experiment 1 and 2 and lower concentrated *S. costatum* inoculations. The initial cell abundance ratio of *S. costatum* to that of *C. didymus* was therefore adjusted to 20:1 (high‐Sc) and 7:1 (low‐Sc). The low‐Sc treatment led to similar initial biomass for both diatoms, when considering different sizes of the cells (Harrison, Conway, Holmes, & Davis, 1977; Menden‐Deuer, Lessard, & Satterberg, 2001) whereas *S. costatum* is dominant in the high‐Sc co‐culturing setups. Lysis of *S. costatum* by *K. algicida* occurred with similar kinetics compared to the above experiments and growth responses were significantly reduced from day 1 after inoculation with the bacteria (*p* ≤ 0.003 for all data points from day 1 onwards at days with equal variance, Table A3). Growth of *C. didymus* did not differ in the high‐ and low‐Sc treatments in the absence of *K. algicida* (at all data points P values exceeded 0.219, Table A3). *K. algicida* strongly modulated the outcome of the co‐culturing. Dependent on the initial *S. costatum* concentration, *C. didymus* growth was either promoted (low‐Sc+Ka) or inhibited (high‐Sc+Ka) in the presence of *K. algicida*. These effects manifested from day 7 onward (day 7: *p* = 0.045, day 10: *p* = 0.029 for growth support) and *p* = 0.017 for growth inhibition at day 11. pH was monitored throughout the co‐culturing and no changes were observed (data not shown). The results support a complex interaction pattern once the three partners can chemically interact.

### 3.4 Effect of co‐culture exudates on the growth of *K. algicida* (Experiment 4)

**Figure 4:**
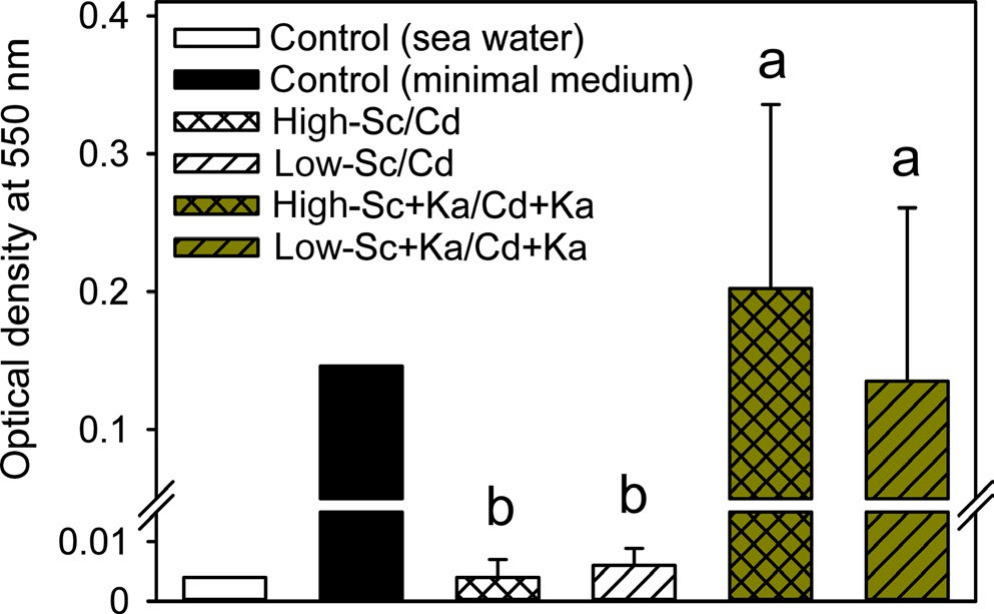
Growth of *K. algicida* in filtered spent medium obtained from the co‐culture systems. Optical density (OD) of *K. algicida* was recorded after eight days in filtrates of the co‐cultures from Figure 3 at day 11. Standard deviation (±*SD*) represents the mean of five replicates except for high‐Sc/Cd (*n* = 3). Means with letters were compared (non‐parametric U‐test) and different letters are significant different (Table A4)

*K. algicida* grew in <0.2 µm filtrates from both treatments of the co‐culture experiment in which bacterial lysis was induced (low‐Sc+Ka/Cd+Ka and high‐Sc+Ka/Cd+Ka) as well as in the minimal medium serving as positive control (Figure 4). However, bacterial cultures after eight days of incubation with the co‐culture filtrates appear whitish whereas *K. algicida* fully gained its typical yellowish phenotype in the minimal medium. Sea water and both <0.2 µm filtrates of co‐culturing experiments that were not infected with *K. algicida* did not support the growth of *K. algicida*.

## 4 DISCUSSION

Only a few studies on algicidal bacteria have focused on their effects on phytoplankton communities (Jung, Kim, Katano, Kong, & Han, 2008; Pokrzywinski, Place, Warner, & Coyne, 2012) and to the best of our knowledge, cascading effects in defined assemblages that give mechanistic insights have not been addressed. Results from this study clearly document that the effect of algicidal bacteria in common bilateral incubation experiments represent an oversimplification compared to the situation in consortia. A cascading effect of algicidal bacteria in mixed algal assemblages can lead to fundamentally different outcomes after bacterial infection compared to mono cultures. In this and previous studies, the pure culture of *C. didymus* was entirely resistant against *K. algicida *(Paul & Pohnert, 2011). In mixed cultures, however, the bacterial lysis of the competitor resulted in concentration dependent effect on the performance of the resistant alga. At high concentration of the lysed competitor growth arrest was observed (Figure 1) while low concentrations of lysed cells supported its growths (Figure 3b). We demonstrate that chemical factors mediate these concentration–dependent interactions in a dynamic manner (Figures 2 and 3). Furthermore we illustrate that the effect of algicidal bacteria and that of allelopathic interactions between two competing algae cannot be fully untangled. Despite the fact that cell densities in our co‐culturing setups exceed those in the natural environment we conclude that overlaying multi–process interaction can also be expected to occur in natural plankton where mixed phyto‐ and bacterioplankton communities prevail. In these natural scenarios close contact interactions with locally enhanced metabolites within the diffusion limited zone (phycosphere) around the producing organism will prevail (Seymour, Amin, Raina, & Stocker, 2017). These steep concentration gradients are not covered in the study here but were averaged over the culture, thus only indirect conclusions about processes in natural waters can be given. Since both diatom genera under investigation frequently dominate phytoplankton blooms and co‐exist and phycospheres will encounter in the oceans, our observations have consequences for natural populations (Kooistra et al., 2008; Li et al., 2017).

A complex interaction network between the three investigated species was observed for mixed cultures in Experiment 1. If both diatoms were inoculated within the same culture vessel the growth *C. didymus* was delayed and *S. costatum* entered the declining phase earlier (Figure 1). Thus, both algae in direct interaction negatively affect the growth of the partner. Allelochemical interactions would be a possible explanation for this phenomenon but microalgae in direct contact might influence the respective growth using alternative mechanisms. These include inhibition by shading effects that reduce light availability to the competitors, concurrent consumption of nutrients, or direct interactions mediated by cell‐cell contact (Dunker, Althammer, Pohnert, & Wilhelm, 2017). If *K. algicida* is added as third interaction partner, fast lysis of *S. costatum* is observed, which is in full agreement with experiments using isolated cultures (Paul & Pohnert, 2011). The lysis indicates that quorum sensing up‐regulation of algicidal activity as observed in Paul and Pohnert (2011) is active in our experimental setups. Interestingly, growth of *C. didymus* that is fully resistant against bacterial lysis in bilateral interactions was entirely suppressed in the tripartite co‐culturing. A possible explanation for this observed inhibition is that exudates, released from declining or perished cells of *S. costatum* are inhibiting the growth of the competitor (Imada, Kobayashi, Tahara, & Oshima, 1991). This is also supported by the observation that the growth arrest by lysed cells is concentration dependent (see below). Alternatively, the bacteria might up‐regulate their algicidal activity in the presence of both partners, thereby resulting in an increased infectivity even against the otherwise resistant algae.

To evaluate if chemical signals meditate this interaction, we conducted Experiment 2 with filtrates of incubations where either the algae or both, algae and bacteria were removed using different pore sized filters (Figure 2). These experiments confirmed that in fact the “smell of death” of the declining or lysed *S. costatum* population is responsible for the observed effect on *C. didymus* (Figure 2c). To exclude potentially overlaying effects of nutrient starvation we exposed freshly transferred *C. didymus* to the filtrates thereby guaranteeing sufficient access to nutrients during three days of the experiment. The inhibitory effect on *C. didymus* was observed in both, filtrates from declining and bacterially lysed *S. costatum* and is thus most likely caused by chemical mediators from this diatom. This main or exclusive contribution of *S. costatum* derived mediators is also supported by the notion that algicidal proteases that are up‐regulated in *S. costatum* infections (Figure 2b) are not affecting *C. didymus* (Figure 2c) (Paul & Pohnert, 2013). Also the role of proteases released during death of diatoms in response to abiotic stress as observed by Berges and Falkowski (1998) can be excluded since no such increase was observed in the declining *S. costatum* culture (Figure 2b). Qualitatively, the *Skeletonema*‐effect is in agreement with previous studies demonstrating growth–inhibiting effects of spent medium of diatoms from the end of their growth phase (Imada et al., 1991; Vidoudez & Pohnert, 2008). Negative influence on co‐occurring phytoplankton has been repeatedly reported for *Skeletonema *spp. (Wang et al., 2017; Yamasaki et al., 2011) while only few reports on positive allelopathy of this alga are known (Paul, Barofsky, Vidoudez, & Pohnert, 2009). The metabolite class of polyunsaturated aldehydes (PUAs) are often brought forward as examples for negative allelopathic exudates of *Skeletonema* sp. (Fontana, d'Ippolito, Cutignano, Miralto et al., 2007; Fontana, d'Ippolito, Cutignano, Romano et al., 2007; Ianora, Bentley et al., 2011; Ianora, Romano et al., 2011; Sieg, Poulson‐Ellestad, & Kubanek, 2011; Wichard et al., 2005). These metabolites are released during the late exponential phase of growth (Vidoudez & Pohnert, 2008) but cell lysis triggers an even more pronounced production (Ribalet et al., 2014). Other compound classes, such as sterol sulfates from *Skeletonema marinoi* can mediate cell death (Gallo, d'Ippolito, Nuzzo, Sardo, & Fontana, 2017). While these sterols have been made responsible for an auto induced cell lysis it can be envisaged that that they might also alter the physiology of other diatoms, particularly when suddenly released after lysis (Xu, Tang, Qin, Duan, & Gobler, 2015). The fact that sterile filtered culture supernatants had consistently lower effects on *C. didymus* compared to 5 µm filtrates that still contained bacteria and other particulate organic matter suggests that in addition to diffusible chemicals additional factors are involved in the interaction (Figure 2c). We did not undertake further elucidation of the involved chemical triggers but rather focused on functional aspects of the interaction.

In direct contact co‐cultures the growth of *C. didymus* was inhibited in a more pronounced way in the presence of *S. costatum* and *K. algicida* compared to the presence of *S. costatum* alone (Figure 1b). In contrast, filtrates from the co‐culture at day 10 suppressed growth less compared to those from declining cultures (Figure 2c). Here, the bacterium apparently reduces the harmful effect, which could be due to recycling of diatom‐derived organic matter during the prolonged time of the experiment or due to nutrient exchange that is often the basis for positive algal/bacterial interactions (Amin, Parker, & Armbrust, 2012; Orellana, Pang, Durand, Whitehead, & Baliga, 2013). It is also possible that *K. algicida *metabolizes toxins released by lysed *S. costatum *within the 10 days required to manifest the effect. Such detoxification by bacteria has already been documented in cyanobacteria (Yamada, Murakami, Kawamura, & Sakakibara, 1994).

To obtain a more refined picture about the mechanism of interaction we carried out a non‐contact co‐cultivation Experiment 3 (Figure 3). In the experimental setup we ensure that chemical mediators can reach all interaction partners within the system, while the two competing algal species remain spatially separated (Paul et al., 2009). At comparable cell concentrations to the experiments described above, exudates from the lysed *S. costatum* resulted in significantly inhibited growth of *C. didymus*. This effect manifested most pronouncedly toward the end of the experiment (Figure 3b). Mediators released from the association of *K. algicida* with the dead dense *S. costatum* culture are thus freely diffusible and have the capacity to reduce *C. didymus* that is otherwise resistant to the lytic bacterium. The pH remained constant throughout the experiments thereby excluding this potential cause for reduced performance of *C. didymus*.

The situation is entirely reversed if *S. costatum* cell counts in the co‐culturing chamber are reduced. In this case, *C. didymus* performance is increased after day 7 higher abundance of this alga is observed compared to the control (Figure 3b). It has been demonstrated for the diatom *Thalassiosira weissflogii* that it performs better in co‐culture with *Skeletonema* sp. and such a support is obviously also occurring in the mixed culture under study in this investigation (Paul et al., 2009). Since *C. didymus* is performing better if *S. costatum* is lysed by the bacterium it might be envisaged that it benefits metabolites or nutrients released from disrupted cells. Use of organic substrates would require the capability of mixotrophic growth that has been documented in diatoms previously (Shishlyannikov, Klimenkov, Bedoshvili, Mikhailov, & Gorshkov, 2014; Villanova et al., 2017). Nutrient release can also be envisaged since diatoms are known to store internally nutrients that might be released during bacterial lysis (De La Rocha, Terbruggen, Volker, & Hohn, 2010). Alternatively, metabolites such as PUA, released during diatom wounding might be priming defence capabilities in the co‐cultured algae, a phenomenon that has been described for PUA treated monocultures of the diatom *Phaeodactylum tricornutum* (Vardi et al., 2006).

Looking at the third interaction partner we could show *K. algicida* benefits from the lysis of the diatoms. By exposing it to sterile filtrates of the co‐cultivations we observed that it grew effectively on exudates from setups with lysed *S. costatum *cells compared to those that contained only the exudates of the co‐cultures without bacterial lysis (Figure 4). From macroscopic investigations of cell pellets of grown bacterial cultures we recognized that cultures after growth on algal‐derived lysis products appear whitish compared to the yellowish controls in artificial bacterial media. Obviously, the algal exudates remaining in the co‐culture are not sufficient for the full development of the phenotype but support efficient growth in the range of that observed in minimum medium. The observed increased bacterial growth on lysis products fully agrees with field data that heterotrophic bacteria follow the bloom of phytoplankton (Teeling et al., 2012).

## 5 CONCLUSION

Despite the already complex outcome of the experiments, the situation in the plankton are undoubtedly more complex, since additional associated microorganism might modulate the chemical mediators by metabolic transformations (Margulis, 1990). However, our study illustrates that the chemical interaction within even a simple model community makes the outcome hard to predict. This calls for caution if algicidal bacteria are released into the environment to control for example harmful algal blooms.

## CONFLICT OF INTERESTS

The authors declare no conflict of interests.

## AUTHORS CONTRIBUTION

GP and AB conceived the study, AB performed the experiments, AB and GP wrote, edited, and approved the manuscript.

## ETHICS STATEMENT

Not required.

## Data Availability

Underlying data for this publication will be made available upon reasonable request.

## APPENDIX 1

**Table A1a:** Cell counts of *S. costatum* in co‐cultures and mono‐cultures ±bacterial infection were compared using *U*‐tests

Comparisons	Size	Median (cells/mL)	25% percentile	75% percentile	T	*p* (exact)
Day1: Sc+Cd versus Sc+Cd+Ka	4;4	150,768;10,837	110,128;10,519	186,628;18,567	26.00	0.029*
Day4: Sc+Cd versus Sc+Cd+Ka	6;7	305,044;4,144	277,153;3,825	339,469;7,969	63.00	0.001**
Day6: Sc+Cd versus Sc+Cd+Ka	4;4	276,356;3,825	224,878;2,948	330,703;9,244	26	0.029*
Day8: Sc+Cd versus Sc+Cd+Ka	6;7	257,231;2,231	195,234;638	319,866;4,781	63	0.001**
Day1: Sc+Ka versus Sc+Cd+Ka	4;4	10,838;10,838	10,519; 10,519	18,567;18,567	18	1
Day4: Sc+Ka versus Sc+Cd+Ka	4;7	9,244;4,144	8,766;3,825	9,961;7,969	36	0.024*
Day6: Sc+Ka versus Sc+Cd+Ka	4;4	5,897;3,825	5,100; 2,948	8,367; 9,244	22	0.343
Day8: Sc+Ka versus Sc+Cd+Ka	4;7	4,941;2,231	3,586;638	5,817;4,781	33	0.109
Day1: Sc versus Sc+Ka	4;4	112,040;10,838	82,397;10,519	129,492; 18,567	26	0.029*
Day4: Sc versus Sc+Ka	4;4	325,125;9,244	258,506;8,766	336,759;9,961	26	0.029*
Day6: Sc versus Sc+Ka	4;4	356,044;5,897	320,344;5,100	417,084;8,367	26	0.029*
Day8: Sc versus Sc+Ka	4;4	391,744;4,941	329,428;3,586	405,769;5,817	26	0.029*
Day1: Sc versus Sc+Cd	4;4	112,040; 150,768	82,397; 110,128	129,492; 186,628	12	0.114
Day4: Sc versus Sc+Cd	4;6	325,125; 305,043	258,506; 277,153	336,759; 339,469	23	0.914
Day6: Sc versus Sc+Cd	4;4	356,043; 276,356	320,344; 224,878	417,084; 330,703	23	0.200
Day8: Sc versus Sc+Cd	4;6	391,743; 257,231	329,428; 195,234	405,769; 319,866	32	0.038*

Note

Level of significances:

* *p* < 0.05

** *p* < 0.01

*** *p* < 0.0001.

**Table A1b:** Cell counts of *C. didymus* in co‐cultures and mono‐cultures ±bacterial infection were compared using U‐tests

Comparisons	Size	Median (cells/ml)	25% percentile	75% percentile	T	*p* (exact)
Day2: Sc+Cd versus Sc+Cd+Ka	6;6	3,028; 2071	1913;0	7,438;5,844	52	0.181
Day4: Sc+Cd versus Sc+Cd+Ka	6;7	6,295; 6,693	5,220;2,550	23,375;16,044	45.5	0.628
Day5: Sc+Cd versus Sc+Cd+Ka	6;7	5,657; 17,956	4,821;13,706	44,758;19,656	34	0.295
Day6: Sc+Cd versus Sc+Cd+Ka	4;4	18,009; 14,343	15,380;10,678	20,639;25,181	21	0.486
Day7: Sc+Cd versus Sc+Cd+Ka	6;7	34,584; 17,850	18,966;13,069	121,603;20,400	55	0.073
Day8: Sc+Cd versus Sc+Cd+Ka	6;7	47,653; 14,343	29,803;6,163	176,083;19,763	63	0.001**
Day2: Cd+Ka versus Sc+Cd+Ka	4;7	3,188;2072	1952;0	3,586;5,844	28	0.527
Day4: Cd+Ka versus Sc+Cd+Ka	4;7	12,511;6,694	9,363;2,550	16,137;16,044	31	0.230
Day5: Cd+Ka versus Sc+Cd+Ka	4;7	27,253;17,956	20,798;13,706	49,486;19,656	35	0.042*
Day6: Cd+Ka versus Sc+Cd+Ka	4;4	66,141;14,344	65,105;10,678	81,759;25,181	26	0.029*
Day7: Cd+Ka versus Sc+Cd+Ka	4;7	131,963;17,850	99,530;13,069	141,684;20,400	38	0.006**
Day8: Cd+Ka versus Sc+Cd+Ka	4;7	188,700;14,344	128,217;6,163	225,755;19,763	38	0.006**
Day2: Cd versus Cd+Ka	4;4	3,585;3,188	2072;1952	4,263;3,586	20.5	0.486
Day4: Cd versus Cd+Ka	4;4	23,348;12,511	18,248;9,363	29,285;16,137	26	0.029*
Day5: Cd versus Cd+Ka	4;4	73,471;27,253	34,664;20,798	92,198;49,486	24	0.114
Day6: Cd versus Cd+Ka	4;4	132,600;66,141	102,080;65,105	174,117;81,759	26	0.029*
Day7: Cd versus Cd+Ka	4;4	192,365;131,963	143,677;99,530	262,809;141,684	24	0.114
Day8: Cd versus Cd+Ka	4;4	148,856;188,700	130,130;128,217	199,378;225,755	16	0.686
Day2: Cd versus Sc+Cd	4;6	3,585;3,028	2072;3,028	4,263;7,438	22	1
Day4: Cd versus Sc+Cd	4;6	23,348;6,295	18,248;5,220	29,285;23,375	29	0.171
Day5: Cd versus Sc+Cd	4;6	73,471;5,657	34,664;4,821	92,198;44,758	32	0.038*
Day6: Cd versus Sc+Cd	4;4	132,600;18,009	102,080;15,380	174,117;20,639	26	0.029*
Day7: Cd versus Sc+Cd	4;6	192,365;34,584	143,677;18,966	262,809;121,603	34	0.010*
Day8: Cd versus Sc+Cd	4;6	148,856;47,653	130,130;29,803	199,378;176,083	28	0.257

Note

Level of significances:

* *p* < 0.05,

** *p* < 0.01,

*** *p* < 0.0001.

**Table A2a:** Comparison of *S. costatum *with and without *K. algicida, *the infection happened at day 0

Comparisons (*S. costatum* vs. S*. costatum *+K*. algicida*)	Size	Mean (RFU)	Std Dev	*t*	*df*	*p*
Day −15	3;3	325;348	18.4;19.9	−1.443	4	0.223
Day −10	3;3	2,656;2,392	631.4;130.8	0.708	4	0.518
Day −5	3;3	7,624;6,156	2,421.4;718.0	1.006	4	0.371
Day −3	3;3	11,876;11,148	369.5;1,441.5	0.846	4	0.445
Day 0	3;3	10,436;9,155	251.4;935.7	2.290	4	0.084
Day 1	3;3	9,320;6,035	550.3;1,367	3.861	4	0.018*
Day 2	3;3	8,556;2,573	318.0;575.8	15.754	4	<0.001***
Day 3	3;3	8,087;949	496.8;72.6	24.622	4	<0.001***
Day 5	3;3	6,858;852	627.2;59.7	16.511	4	<0.001***
Day 7	3;3	5,107;703	616.3;72.1	12.293	4	<0.001***
Day 10	3;3	3,747;499	526.4;77.1	10.572	4	<0.001***

Note

Values are based on *in vivo* chlorophyll *a* fluorescence (RFU). T test performed with two‐tailed *p*‐values.

Level of significances:

* *p* < 0.05,

** *p* < 0.01,

*** *p* < 0.0001.

**Table A2b:** Comparison of protease activity in relative fluorescence units

ANOVA				Post hoc test		
	Size	Mean	Std Dev		*t*	*p*
Filtrates of day 0 (*df* = 7; *F* = 2.967; *p* = 0.141)				‐		
*S. costatum*	3	539	483.9			
*S. costatum* +K*. algicida*	3	1,134	640.4			
*K. algicida* in sea water	2	2,142	1,145.5			
Filtrates of day 5 (*df* = 8; *F* = 70.613; *p* < 0.001)				Holm‐Sidak (all pairwise) α 0.05		
*S. costatum*	3	851	738.5	*S. costatum* versus *S. costatum *+K*. algicida*	11.5	<0.001***
*S. costatum* +K*. algicida*	3	12,057	1897.6	*S. costatum *+K*. algicida* versus *K. algicida* in sea water	8.334	<0.001***
*K. algicida* in sea water	3	3,939	352.5	*S. costatum* versus *K. algicida* in sea water	3.170	0.019*
Filtrates of day 10 (*df* = 8; *F* = 36.111; *p* < 0.001)				Holm‐Sidak (all pairwise) α 0.05		
*S. costatum*	3	1,064	1,226.6	*S. costatum* versus *S. costatum *+K*. algicida*	8.473	<0.001***
*S. costatum* +K*. algicida*	3	7,188	636.6	*S. costatum *+K*. algicida* versus *K. algicida* in sea water	4.810	0.006**
*K. algicida* in sea water	3	3,712	664.4	*S. costatum* versus *K. algicida* in sea water	3.663	0.011*

Note

Averaged blank values for protease activity of sea water were subtracted before one way analysis of variance (ANOVA) was carried out. Background subtraction resulted in one negative value (*S. costatum* with day 5 filtrates) which was set to zero.

Level of significances:

* *p* < 0.05,

** *p* < 0.01,

*** *p* < 0.0001.

**Table A2c:** Comparison of *C. didymus *growth response with filtrates of declining *S. costatum *and bacterial‐lysed *S. costatum *obtained at three time points based on *in vivo* chlorophyll *a* fluorescence values (RFU)

Comparisons (*S. costatum* vs. S*. costatum*+K*.algicida*)	Size	Mean (RFU)	Std Dev	*t*	*df*	*p*
Day0 filtrate: 0.2 µm (lysed) versus 5 µm (lysed)	3;3	104;52	8.2;14.6	5.366	4	0.006**
Day5 filtrate: 0.2 µm (lysed) versus 5 µm (lysed)	3;3	81;15	4.1;6.4	15.008	4	<0.001***
Day10 filtrate: 0.2 µm (lysed) versus 5 µm (lysed)	3;3	84;14	0.9;1.7	62.645	4	<0.001***
Day0 filtrate: 0.2 µm (declined) versus 5 µm (declined)	3;3	80;53	2.6;4.8	8.610	4	0.001**
Day5 filtrate: 0.2 µm (declined) versus 5 µm (declined)	3;3	75;14	4.8;4.2	16.646	4	<0.001***
Day10 filtrate: 0.2 µm (declined) versus 5 µm (declined)	3;3	49;−7	1.3;7.4	12.821	4	<0.001***
Day0 filtrate: 0.2 µm (declined) versus 0.2 µm (lysed)	3;3	80;104	2.6;8.2	−4.874	4	0.008**
Day5 filtrate: 0.2 µm (declined) versus 0.2 µm (lysed)	3;3	75;81	4.8;4.1	−1.497	4	0.209
Day10 filtrate: 0.2 µm (declined) versus 0.2 µm (lysed)	3;3	49;84	1.3;0.9	−38.224	4	<0.001***
Day0 filtrate: 5 µm (declined) versus 5 µm (lysed)	3;3	53;52	4.8;14.6	0.0844	4	0.937
Day5 filtrate: 5 µm (declined) versus 5 µm (lysed)	3;3	14;15	4.2;6.4	−0.141	4	0.895
Day10 filtrate: 5 µm (declined) versus 5 µm (lysed)	3;3	−7;14	7.4;1.7	−4.649	4	0.0097**

Note

T test performed with two‐tailed *p*‐values.

Level of significances:

* *p* < 0.05,

** *p* < 0.01,

*** *p* < 0.0001.

**Table A3:** Comparison of diatom growth response in non‐contact co‐cultures (± bacterial infections) were compared using *t* tests. Significances were obtained as *p*‐values from two‐tailed tests

	Size	Mean (RFU)	Std Dev	*T*	*df*	*p*–value
*S. costatum* (low‐Sc/Cd); *S. costatum* (low‐Sc+Ka/Cd+Ka)						
Day 1	5;5	1,803; 1,457	96; 96	5.683	8	<0.001***
Day 2	5;5	2,480; 1,664	323; 179	4.935	8	0.001**
Day 3	5;5	3,467; 1,441	308; 297	10.580	8	<0.001***
Day 4#	5;5	4,712;1,060	560.5;354.1	12.318	8	<0.001***
*S. costatum* (high‐Sc/Cd); *S. costatum* (high‐Sc+Ka/Cd+Ka)						
Day 1	5;5	4,180; 3,486	320; 191	4.169	8	0.003**
Day 2	5;5	5,291; 3,669	511; 182	6.687	8	<0.001***
Day 3#	5;5	7,499; 2,947	748; 224	13.032	8	<0.001***
*C. didymus* (low‐Sc/Cd); *C. didymus* (high‐Sc/Cd)						
Day 1	5;5	1,847; 1,628	321; 228	1.243	8	0.249
Day 2	5;5	1,878; 1,824	558; 504	0.159	8	0.878
Day 3	5;5	2,700; 2,438	889; 1,271	0.378	8	0.715
Day 4	5;5	3,887; 3,388	1,090; 1,485	0.606	8	0.561
Day 6	5;5	8,024; 6,896	2,614; 2,225	0.735	8	0.483
Day 7	5;5	9,352; 8,827	2,867; 2,442	0.312	8	0.763
Day 8	5;5	10,486; 9,806	2,456; 2,480	0.436	8	0.675
Day 9	5;5	11,459; 11,141	2,506; 2,111	0.217	8	0.834
Day 10	5;5	12,820; 11,604	2,585; 1625	0.890	8	0.399
Day 11	5;5	15,381; 13,285	2,738; 2,200	1.334	8	0.219
*C. didymus* (low‐Sc/Cd); *C. didymus* (low‐Sc+Ka/Cd+Ka)						
Day 1	5;5	1,847;1,752	321;213	0.552	8	0.596
Day 2	5;5	1,878;1,875	558;578	0.007	8	0.995
Day 3	5;5	2,700;2,586	889.1;904.9	0.201	8	0.846
Day 4	5;5	3,887;3,608	1,090;596	0.503	8	0.628
Day 6	5;5	8,024;9,249	2,614;1549	−0.902	8	0.394
Day 7	5;5	9,352;12,870	2,867;1657	−2.376	8	0.045*
Day 8	5;5	10,486;12,957	2,456.4;3,002.9	−1.424	8	0.192
Day 9	5;5	11,459; 14,676	2,506; 2,405	−2.071	8	0.072
Day 10	5;5	12,820;17,044	2,584.7;2,425.7	−2.665	8	0.029*
Day 11	5;5	15,381; 12,939	2,738;10,284	0.513	8	0.622
*C. didymus* (high‐Sc/Cd); *C. didymus* (high‐Sc+Ka/Cd+Ka)						
Day 1	5;5	1,628;1,619	228.2;166.5	0.0735	8	0.943
Day 2	5;5	1,824;1,800	504.4;603.5	0.0685	8	0.947
Day 3	5;5	2,438;2,371	1,270.8;1,204.2	0.0086	8	0.934
Day 4	5;5	3,388;3,179	1,485.4;1,498.0	0.222	8	0.830
Day 6	5;5	6,895.9;6,038	2,224.8;2,951.1	0.519	8	0.618
Day 7	5;5	8,827;7,845	2,442.2;3,710.1	0.494	8	0.634
Day 8	5;5	9,806;7,908.8	2,479.6;4,031.0	0.897	8	0.396
Day 9	5;5	11,141;8,250	2,111.3;2,364.8	2.040	8	0.076
Day 10	5;5	11,604;8,243	1624.5;4,930.7	1.448	8	0.186
Day 11	5;5	13,285;4,205	2,199.9;6,405.3	2.998	8	0.017*

Note

Level of significances:

^#^ No homogeneity of variance was observed after these days.

* *p* < 0.05,

** *p* < 0.01,

*** *p* < 0.0001.

**Table A4:** Differences in growth of *K. algicida *in filtered spent medium obtained from co‐cultures evaluated by U‐tests (Mann–Whitney Rank Sum Test)

Comparisons	Size	Median (cells/ml)	25% percentile	75% percentile	T	*p* (exact)
high‐Sc+Ka/Cd+Ka versus low‐Sc+Ka/Cd+Ka	5;5	0.220;0.145	0.090;0.011	0.305;0.255	33	0.310
high‐Sc+Ka/Cd+Ka versus high‐Sc/Cd	5;3	0.220;0.004	0.090;0.001	0.305;0.007	6	0.036*
high‐Sc+Ka/Cd+Ka versus low‐Sc/Cd	5;5	0.220;0.005	0.090;0.004	0.305;0.008	40	0.008**
low‐Sc+Ka/Cd+Ka versus high‐Sc/Cd	5;3	0.145;0.004	0.011;0.001	0.255;0.007	6	0.036*
low‐Sc+Ka/Cd+Ka versus low‐Sc/Cd	5;5	0.145;0.005	0.011;0.004	0.255;0.008	38.5	0.016*
high‐Sc/Cd versus low‐Sc/Cd	3;5	0.004;0.005	0.001;0.004	0.007;0.008	11	0.571

Note

Level of significances:

* *p* < 0.05,

** *p* < 0.01,

*** *p* < 0.0001.
